# Supplementary material for: Are Graduate Medical Trainees Prepared for the Personalized Genomic Medicine Revolution? Trainee Perspectives at One Institution
Source: J Pers Med. 2023 Jun 21;13(7):1025. doi: 10.3390/jpm13071025 (PMC10381337; doi:10.3390/jpm13071025)
Supplement: Supplementary file 1 [file jpm-13-01025-s001.zip › jpm-2397012-supplementary.pdf]

Local Survey of Resident & Fellow Knowledge, Skills & Attitudes toward Personalized Medicine & Pharmacogenetics

The following survey is being conducted with the goal to assess graduate medical trainee knowledge, skills and, attitudes towards personalized medicine and pharmacogenetics.
The survey will take ~ 15 minutes to complete.

First, we are going to ask about your knowledge of **personalized medicine**. In this survey, personalized medicine (also known as precision medicine) refers to a type of medical care in which disease screening and/or treatment are customized for an individual patient, typically based on his or her genetics.

How much do you agree or disagree with each of the following statements?

	Strongly agree	Agree	Disagree	Strongly disagree
1. I feel sufficiently informed about personalized medicine.	❒	❒	❒	❒
2. My patients ask about personalized medicine.	❒	❒	❒	❒

Next, we are going to ask about your knowledge of disease genetics and pharmacogenetics.
For the purpose of this survey, **disease genetics** refers to genetic variation known to impact disease risk. **Disease genetic testing** refers to testing used to evaluate disease risk or carrier status. For example, *BRCA1* is a gene known to confer increased risk of breast and ovarian cancer.
Pharmacogenetics (also known as pharmacogenomics) refers to how a patient's genetics impacts drug metabolism and/or response. **Pharmacogenetic testing** refers to testing used to predict a patient's response to medication therapy.

How much do you agree or disagree with each of the following statements?

	Strongly agree	Agree	Disagree	Strongly disagree
3. I am confident in my knowledge about the influence of genetics on *disease risk.*	❒	❒	❒	❒
4. I am confident in my knowledge about the influence of genetics on *disease screening*.	❒	❒	❒	❒
5. I am confident in my ability to interpret a *disease genetic test* result.	❒	❒	❒	❒
6. I am confident in my ability to explain a *disease genetic test* result to a patient.	❒	❒	❒	❒
7. My patients ask about *disease genetic* testing.	❒	❒	❒	❒
8. There is sufficient evidence to support ordering *disease genetic tests.*	❒	❒	❒	❒
9. I am confident in my knowledge about the influence of genetics on *medication therapy*.	❒	❒	❒	❒
10. I am confident in my ability to interpret a *pharmacogenetic test* result.	❒	❒	❒	❒
11. I am confident in my ability to explain a *pharmacogenetic test* result to a patient.	❒	❒	❒	❒
12. My patients ask about *pharmacogenetic* testing.	❒	❒	❒	❒

13. There is sufficient evidence to support ordering *pharmacogenetic tests.*	❐	❐	❐	❐

14. In which of the following settings have you received education or training in personalized medicine, including disease genetics and pharmacogenetics? *Select all that apply.*
   - ❐ Undergraduate school
   - ❐ Medical or graduate school
   - ❐ Residency training
   - ❐ Fellowship training
   - ❐ Grand Rounds or other lectures
   - ❐ Continuing medical education (CME) program
   - ❐ Academic or research conference
   - ❐ Institutional education or training initiatives
   - ❐ Other (please specify) *(paragraph box pops up)*
   - ❐ I have not received any education or training in personalized medicine

15. Which of the following resources have you used for information about pharmacogenetic testing? *Select all that apply.*
   - ❐ Clinical decision support tool in the electronic medical record
   - ❐ Consultations with experts or peer discussions
   - ❐ National guidelines
   - ❐ FDA drug label information
   - ❐ Scientific literature
   - ❐ Other internal or external resources/tutorials
   - ❐ I have not used any resources for information about pharmacogenetic testing

Please indicate your level of awareness and use of the following resources.

	I am NOT aware of this resource	I am aware of this resource	I have used this resource in clinical practice
16. Clinical Pharmacogenetics Implementation Consortium (CPIC) Guidelines	❐	❐	❐
17. Pharmacogenomics Knowledge Base (PharmGKB)	❐	❐	❐
18. Dutch Pharmacogenetics Working Group Guidelines	❐	❐	❐
19. Clinical Genome Resource (ClinGen)	❐	❐	❐
20. ClinVar	❐	❐	❐
21. American College of Medical Genetics (ACMG)	❐	❐	❐
22. Implementing Genomics in Practice (IGNITE) Spark toolbox	❐	❐	❐

23. How often **in the past 12 months** have you ordered any type of genetic test (including disease genetics and pharmacogenetics) for a patient?
   - ❐ 0 times
   - ❐ 3-10 times
   - ❐ More than 25 times
   - ❐ 1-2 times
   - ❐ 11-25 times
   - ❐ Unsure/Don’t know

24. [*If ‘0 times’ is selected for Q23, skip to Q25.*] In the past 12 months, which of the following *pharmacogenetic* tests have you ordered in the clinical setting? *Select all that apply.*
   - ❐ Single-gene testing (examples include *TPMT*, *CYP2C19*, *CYP2D6* genotyping)

❒ Multi-gene panel-based testing (e.g., Genesight, OneOme, Genomind)
❒ Other (please specify) *(pop up box appears for comments)*

25. Which of the following is a barrier to *disease genetic testing* in your clinical practice? *Select all that apply.*
❒ Uncertain about the clinical value of the test
❒ Cost to the patient
❒ Don't know what test to order
❒ Don't know how to interpret test results
❒ Turnaround time/delay in treatment
❒ Not applicable for my patients
❒ Ethical or privacy concerns
❒ Other (please specify) *(pop up box appears for comments)*
❒ None, there are no barriers to genetic testing in my clinical practice.

26. Which of the following is a barrier to *pharmacogenetic testing* in your clinical practice? *Select all that apply.*
❒ Uncertain about the clinical value of the test
❒ Cost to the patient
❒ Don't know what test to order
❒ Don't know how to interpret test results
❒ Turnaround time/delay in treatment
❒ Not applicable for my patients
❒ Ethical or privacy concerns
❒ Other (please specify) *(pop up box appears for comments)*
❒ None, there are no barriers to genetic testing in my clinical practice.

Now we are going to ask questions about your perspective on the clinical utility of both disease genetic and pharmacogenetic testing and its impact on clinical decision making in your practice setting.

- **Disease genetics** refers to genetic variation known to impact disease risk.
- **Pharmacogenetics** refers to how a patient's genetics impacts drug metabolism and/or response.
- The field of Personalized Medicine includes both disease genetics and pharmacogenetics.

Clinical utility has been defined by some experts as "the relevance and usefulness of an intervention in patient care." It has also been defined as "a test's health-care value." Please keep these definitions in mind as you respond to each item.

How much do you agree or disagree with the following statements?

	**Strongly agree**	**Agree**	**Disagree**	**Strongly disagree**
27. Knowledge of a patient's *disease genetics* would likely influence clinical decision making.	❒	❒	❒	❒
28. Knowledge of a patient's *disease genetics* would likely lead to improved clinical outcomes.	❒	❒	❒	❒
29. All things considered, the benefits of *disease genetic* testing likely outweigh the risks or costs.	❒	❒	❒	❒
30. I would still perform a *disease genetic* test even if the results were difficult to interpret.	❒	❒	❒	❒
31. Knowledge of a patient's *pharmacogenetics* would likely lead to improved clinical outcomes.	❒	❒	❒	❒

32. *Pharmacogenetic*-guided therapy is likely better than standard of care/clinically-guided therapy.	❒	❒	❒	❒
33. All things considered, the benefits of *pharmacogenetic* testing likely outweigh the risks or costs.	❒	❒	❒	❒
34. I would still perform a *pharmacogenetic* test even if the results were difficult to interpret.	❒	❒	❒	❒
35. My clinical program would be able to integrate *personalized medicine* into its workflow.	❒	❒	❒	❒
36. I would find personalized medicine successful if I see that it leads to a change in clinical management of my patients.	❒	❒	❒	❒

37. When new ideas are introduced, clinicians, like other professionals, may be categorized on a scale of innovativeness. *Please select one category which best describes you.*
    - ❒ Bound by tradition, very skeptical of change
    - ❒ Skeptical of change, only adopt an innovation after it has been tried by the majority
    - ❒ Adopt new ideas before the average person, but need to see evidence of success before adopting
    - ❒ Aware of the need to change and very comfortable adopting new ideas
    - ❒ Want to be the first person to try an innovation

Please answer the following questions about your educational needs.

38. If you were to receive education about personalized medicine, disease genetics, and/or pharmacogenetics, in what format would you like to receive this education? *Select all that apply.*
    - ❒ Brief online modules
    - ❒ In-person lectures
    - ❒ Written materials
    - ❒ Other (please specify) *(pop up box appears for comments)*

39. How long would you be willing to spend learning about personalized medicine, disease genetics, and/or pharmacogenetics given the current demands on your time? *Choose the one best option.*
    - ❒ Less than 1 hour
    - ❒ 1-2 hours
    - ❒ 2-3 hours
    - ❒ 3-4 hours
    - ❒ More than 4 hours

Finally, please fill out the following information about yourself and your institution.

40. What is your current role? *(select all that apply)* ❒ Resident ❒ Chief Resident ❒ Fellow

41. What is your current post graduate level?
    [*Provide dropdown (PGY1 – PGY12)*]

42. Which institution houses your training program? *Please select one.* ❒ University Hospital/UCHealth ❒ Children's Hospital Colorado ❒ Denver VA ❒ Denver Health ❒ Swedish ❒ Other (please specify) *(fill in area pops up)*

43. Which training program are you currently completing? *Please select one. List alphabetically. Branching logic from above.*
Resident Options: ❒ Anesthesiology ❒ Child & Adolescent Psychiatry ❒ Dermatology ❒ Emergency Medicine ❒ Family Medicine ❒ Family Medicine – Rural Track ❒ General Surgery ❒ Internal Medicine ❒ Internal Medicine – Pediatrics ❒ Medical Genetics & Genomics ❒ Neurology ❒ Neurological Surgery ❒ Obstetrics & Gynecology ❒ Ophthalmology ❒ Orthopedic Surgery ❒ Otolaryngology ❒ Pathology ❒ Pediatrics ❒ Pediatrics – Physical Medicine & Rehabilitation ❒ Physical Medicine & Rehabilitation ❒ Plastic Surgery ❒ Preventive Medicine – Occupational ❒ Psychiatry ❒ Public Health & Preventive Medicine ❒ Radiation Oncology ❒ Radiology – Diagnostic ❒ Radiology – Interventional ❒ Urology ❒ Other (please specify) *(pop up box appears for comments)*
Fellow Options: ❒ Adult ❒ Pediatric ❒ Both
Adult Options: ❒ Addiction Medicine ❒ Addiction Psychiatry ❒ Advanced Heart Failure & Transplant Cardiology ❒ Allergy & Immunology ❒ Cardiothoracic Anesthesiology ❒ Cardiovascular Disease ❒ Clinical Cardiac Electrophysiology ❒ Congenital Cardiac Surgery ❒ Congenital Heart Disease ❒ Consultation-Liaison Psychiatry ❒ Cytopathology ❒ Dermatopathology ❒ Endocrinology, Diabetes & Metabolism ❒ Epilepsy ❒ Forensic Pathology ❒ Forensic Psychiatry ❒ Gastroenterology ❒ Geriatric Medicine ❒ Gynecologic Oncology ❒ Hand Surgery ❒ Hematology/Oncology ❒ Hematopathology ❒ Hospice & Palliative Medicine ❒ Infectious Disease ❒ Interventional Cardiology ❒ Maternal-Fetal Medicine ❒ Medical Genetics & Genomics ❒ Micrographic Surgery & Dermatologic Oncology ❒ Neuromuscular Medicine ❒ Neuroradiology ❒ Nephrology ❒ Orthopedic Spinal Surgery ❒ Orthopedic Sports Medicine ❒ Pain Medicine ❒ Pulmonary Disease & Critical Care Medicine ❒ Radiology – Interventional ❒ Radiology – Neurointerventional ❒ Reproductive Endocrinology & Infertility ❒ Sleep Medicine ❒ Spinal Cord Injury ❒ Sports Medicine ❒ Surgical Critical Care ❒ Thoracic Surgery ❒ Transfusion Medicine ❒ Transplant Hepatology ❒ Trauma & Acute Care ❒ Vascular Neurology ❒ Vascular Surgery ❒ Other (please specify) *(pop up box appears for comments)*
Pediatric Options: ❒ Adolescent Medicine ❒ Allergy & Immunology ❒ Anesthesiology ❒ Cardiology ❒ Child Abuse ❒ Critical Care Medicine ❒ Dermatology ❒ Developmental – Behavioral ❒ Emergency Medicine ❒ Endocrinology ❒ Epilepsy ❒ Gastroenterology ❒ Hematology/Oncology ❒ Hospice & Palliative Medicine ❒ Hospital Medicine ❒ Infectious Disease ❒ Medical Genetics & Genomics ❒ Neonatal-Perinatal Medicine ❒ Nephrology ❒ Neurology ❒ Orthopedic Surgery ❒ Otolaryngology ❒ Pathology ❒ Physical Medicine & Rehabilitation ❒ Pulmonary Medicine ❒ Radiology ❒ Sleep Medicine ❒ Sports Medicine ❒ Surgery ❒ Urology ❒ Other *(pop up box appears for comments)*
Both Options: ❒ Allergy & Immunology ❒Dermatology ❒ Medical Genetics & Genomics ❒ Neurology ❒ Radiology – Interventional ❒ Sports Medicine ❒ Other (please specify) *(pop up box appears for comments)*

44. What is your gender? ❒ Male ❒ Female ❒ Transgender ❒ Prefer not to answer

45. Which of the following ranges contains your age?
❒ 21-30 years
❒ 31-40 years
❒ 41-50 years
❒ 51-60 years
❒ 61-70 years
❒ More than 70 years

46. What is your race? Select all that apply.
❒ American Indian or Alaska Native
❒ Asian
❒ Black or African American
❒ Native Hawaiian or Other Pacific Islander
❒ White
❒ Other (please specify) (*pop up box appears for comments*)
❒ Prefer not to answer

47. What is your ethnicity? ❒ Hispanic ❒ Non-Hispanic ❒ Prefer not to answer
